# Transcriptome analysis identified aberrant gene expression in pollen developmental pathways leading to CGMS in cotton (*Gossypium hirsutum L*.)

**DOI:** 10.1371/journal.pone.0218381

**Published:** 2019-06-24

**Authors:** Rasmieh Hamid, Hassan Marashi, Rukam S. Tomar, Saeid Malekzadeh Shafaroudi, Pritesh H. Sabara

**Affiliations:** 1 Department of Biotechnology and Plant Breeding, Ferdowsi University of Mashhad, Mashhad, Iran; 2 Department of Biotechnology and Biochemistry, Junagadh Agricultural University, Junagadh, Gujarat, India; 3 Department of Animal Biotechnology, Anand Agricultural University, Anand, Gujarat, India; North Eastern Regional Institute of Science and Technology, INDIA

## Abstract

Male sterility (induced or natural) is a potential tool for commercial hybrid seed production in different crops. Despite numerous endeavors to understand the physiological, hereditary, and molecular cascade of events governing CMS in cotton, the exact biological process controlling sterility and fertility reconstruction remains obscure. During current study, RNA-Seq using Ion Torrent S5 platform is carried out to identify ‘molecular portraits’ in floral buds among the Cytoplasmic Genic Male Sterility (CGMS) line, its near-isogenic maintainer, and restorer lines. A total of 300, 438 and 455 genes were differentially expressed in CGMS, Maintainer, and Restorer lines respectively. The functional analysis using AgriGo revealed suppression in the pathways involved in biogenesis and metabolism of secondary metabolites which play an important role in pollen and anther maturation. Enrichment analysis showed dearth related to pollen and anther’s development in sterile line, including anomalous expression of genes and transcription factors that have a role in the development of the reproductive organ, abnormal cytoskeleton formation, defects in cell wall formation. The current study found aberrant expression of *DYT1*, *AMS* and cytochrome P450 genes involved in tapetum formation, pollen development, pollen exine and anther cuticle formation associated to male sterility as well as fertility restoration of CGMS. In the current study, more numbers of DEGs were found on Chromosome D05 and A05 as compared to other chromosomes. Expression pattern analysis of fourteen randomly selected genes using qRT-PCR showed high concurrence with gene expression profile of RNA-Seq analysis accompanied by a strong correlation of 0.82. The present study provides an important support for future studies in identifying interaction between cyto-nuclear molecular portraits, to accelerate functional genomics and molecular breeding related to cytoplasmic male sterility studies in cotton.

## Introduction

Cotton, (*Gossypium* spp.) belonging to the Malvaceae family, that is an important cash crop because of being a natural source of fiber for various industrial use. In addition to this, cotton is a major source of oil for human consumption and cottonseed meal provides important protein nutrients as animal feed [1]. It provides more than 40% of world’s raw fiber for industrial use in more than 100 countries of temperate, tropical and subtropical regions, truly bestowed the name “white gold” [2]. Plant breeders of India and China [3] are able to increase yield of cotton from 10% to 20% by successfully acceptable limit of exploit heterosis using selective breeding [4]. Most of these heterosis are obtained through quite laborious, tedious, and costly process of artificial emasculation. This hybrid seed production system is practically difficult in application, as the pollen may not be completely removed. This prompted scientists to use a novel technology called ‘male sterility’, as a tool for commercial hybrid seed industry, as it escape the need for hand emasculation in hybrid seed production [5].

Within nature, it was observed that certain hermaphroditic angiosperm species frequently lost their capacity to produce viable pollen grains. The complex trait, termed as ‘cytoplasmic male sterility’ (CMS) is influenced by patterns of mitochondrial genome evolution, as well as intergenomic gene transfer among the organelle and nuclear compartments of plant cells [6, 7]. A three-line technique (Male sterile line ‘A’, maintainer line ‘B’ and restorer line ‘R’) is popularly utilized in the CMS-based production of hybrids. Three-line hybrid production based method has been commonly exploited to produce commercial hybrids in crops like corn, turnip, and rapeseed [8]. However, the global gene expression profiling of CMS and it’s interaction with restorer genes are still unknown to unveil the mechanisms underlying these processes still remains vague. Development of next-generation sequencing technology provides the biggest breakthrough to the scientific community at genomic, transcriptomic and clinical research areas. Genome sequencing of *Gossypium arboretum*, *G*. *raimondii* and G. *hirsutum*, set a stage and provide the backbone to dig deep inside the mechanism behind male sterility in cotton [9].

*Gossypium hirsutum*, with an estimated genome size of 2.25–2.43Gb, is an allotetraploid (AADD, 2n = 4x = 52) upland cotton, which is accounted for more than ninety percent of world's commercial cultivation [10]. The RNA sequencing (RNA-Seq) technology provides unrivalled aspects of messenger RNA profiling with in the cells without any specific prior knowledge [11]. Currently, transcriptome profiling using NGS has been extensively utilized to identify differentially expressed genes (DEGs) in CMS lines of many economically important plant species like, cotton [12], Welsh Onion [13], kenaf [14], cabbage [11], pepper [15] and tomato [16]. However, there are only a few reports of NGS based transcriptome analysis to understand the cytoplasmic male sterility in G. *hirsutum* lines.

In previous study, a preliminary sketch of comparative transcriptome profile between fertile and sterile lines of cotton was done using low depth of RNA sequencing to identify DEGs participating in pathways related to sterility in cotton [17]. The current study is focused on better understanding and identification of ‘molecular portraits’ within DEGs using RNA-Seq by the Ion Torrent S5 technology in floral buds of restorer, sterile and its recurrent parent i.e., the maintainer line from three different cotton lines.

## Materials and methods

### Plant materials

The three lines of cotton (*Gossypium hirsutum* L.) plants i.e. CGMS (JS178), maintainer (JB178), and restorer (JR178) were cultivated during Aug-Oct, 2017 at the Department of Agricultural Biotechnology, Junagadh Agricultural University (JAU), Gujarat, India. The experimental is carried out in a two greenhouse plots with medium black soils falling in the tropical zone, characterized by fairly hot summer, moderately cold winter and humid monsoon. Geographically the experimental site was situated at 21^0^ 49.68’N latitude 70^0^ 44.55 E longitude and at altitude of 97 meters above mean sea level. The CGMS lines using in this study was possessing *G*. *harknessii* cytoplasm and developed by JAU, Gujarat. The CGMS hybrid variety of JS178 is popular because it’s extra-long staple (28 mm MHL), spun up to 69s with ginning 33% and cultivated in Saurashtra region of Gujarat state. All the lines were cultivated in a greenhouse with control environment at 72% relative humidity with a 21/16°C (14 h/ 10 h) day/night temperature regime. After sprouting and growing in greenhouse plots, the flower buds were collected during 37 to 42 days at sporogenous cells stage (SS) and microsporocyte stage (MS) stage of 1.5–2.2 mm and 2.2–2.6 mm sized two developmental stages, respectively. A total of thirty plants from each line, were collected in duplicates (fifteen plants from each plot) and combined to reduce between plant variations. The outer protective portion of the bud was removed carefully and the pooled materials were stored in liquid nitrogen to extract RNA [18].

### Total RNA extraction, m-RNA isolation and c-DNA library preparation

A total RNA was extracted from flower buds of CGMS and its fertile lines anthers at different stages using the “Hot Borate RNA isolation protocol” [19]. The contaminating genomic DNA was removed by DNAseI (Fermentas, Harover, MD) treatment at 37°C for 1 h. The concentration of each RNA sample was measured using ND1000 (Thermo Fisher, Wilmington, DE) spectrophotometer. The quality of the RNA was assessed on 1.0% denaturing agarose gel in combination with the RNA 6000 Pico Kit of Bioanalyzer 2100 (Agilent Technologies, Palo Alto, CA). Total RNA with high purity and integrity (> 7.0 RIN) was utilized for mRNA purification after pooling duplicates in equal quantity. Equimolar pooling of RNA for duplicated samples of all three lines was done. The mRNA purification was performed using poly-(T) oligo-attached magnetic of Dynabeads mRNA DIRECT Purification Kit following the manufacturer's instructions. Quality and quantity of mRNA was confirmed using Qubit spectrofluorometric method. Libraries were generated by using RNA Library Prep Kit (Ion total RNA Seq V2), clonal amplification was carried out to generate multiple copies of fragments on the Ion Sphere (IPS) beads using the Ion torrent OT2 machine and RNA sequencing of each IPS was carried out in Ion Torrent S5 sequencer system using 540 chips.

### Data analysis to identify genes of differential expression

**Fig 1:**
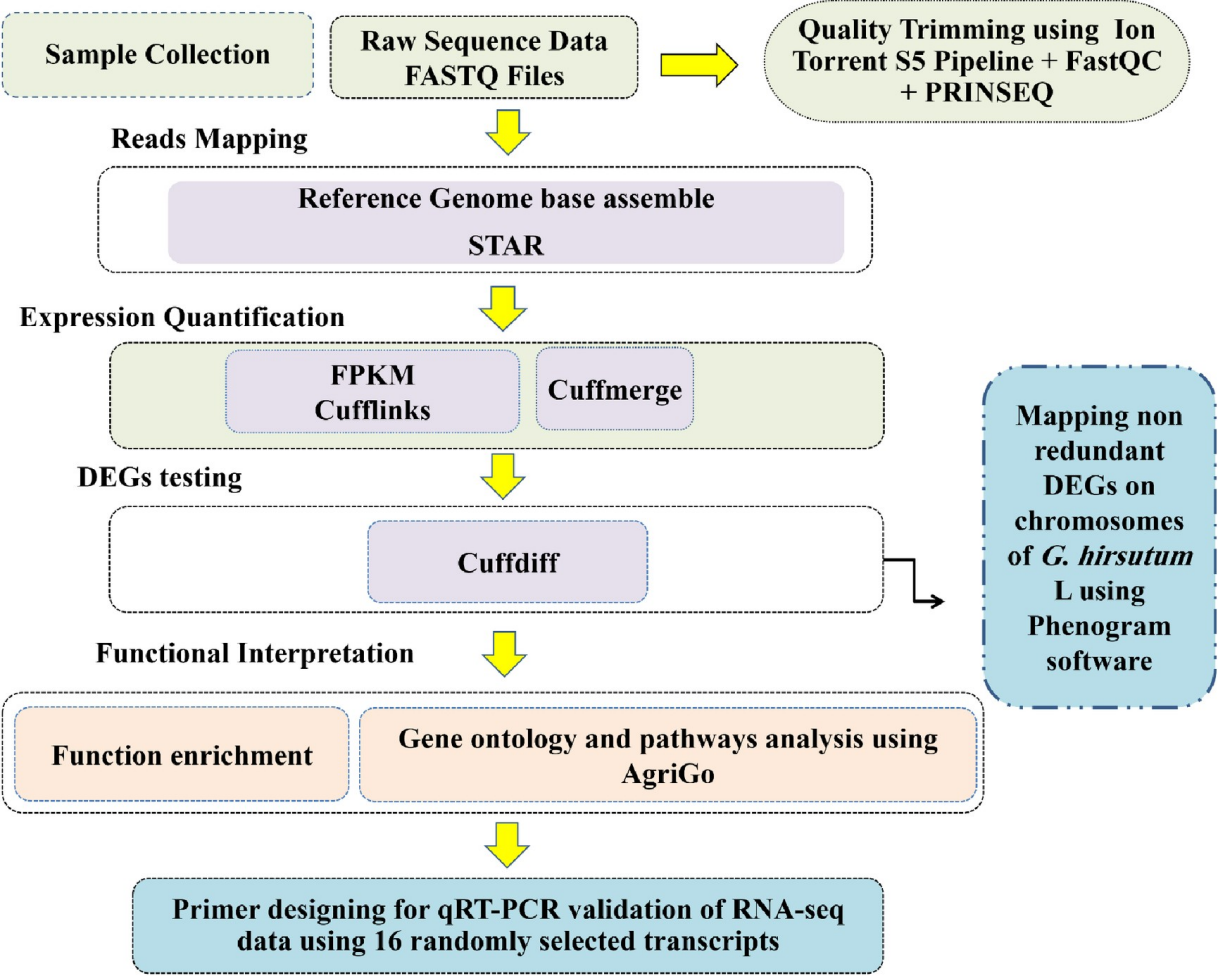
A detailed workflow performed in identification of DEGs in CGMS, maintainer and restorer lines of *Gossypium hirsutum* L.

The data analysis was done as depicted in Fig 1. The mapping was done using the Cotton (*Gossypium hirsutum* L.) reference genome (http://mascotton.njau.edu.cn/info/1054/1118.htm), using STAR 2.5.1 software [20]. The expression levels of genes in terms of fragments per kilobase of transcript per million fragments mapped (FPKM) using Cufflinks [21] were measured in three lines. DEGs between three lines were identified using the ‘Cuffdiff'‘ module with absolute log2 fold change >2 between different groups and a false discovery rate (FDR) of p <0.05 [22]. Location of the differentially expressed gene on Cotton (*Gossypium hirsutum* L.) genome is plotted using Phenogram package of R [23].

### Gene Ontology (GO), pathway and Singular Enrichment Analysis (SEA)

The transcripts that showed significant differential expression were further analyzed by Singular Enrichment Analysis (SEA) to identify enriched Gene Ontology (GO) term using AgriGO v2.0 analysis tools (http://bioinfo.cau.edu.cn/agriGO/) [24]. GO analysis was performed for functional categorization of identified DEGs. The specific biological functions were predicted using the Kyoto Encyclopedia of Genes and Genomes (KEGG) pathway analysis, by mapping the DEGs into the KEGG database. A corrected p ≤0.01 was set as a threshold to identify GO significant enrichment of gene while q ≤0.01 was selected as a threshold of pathway significant enrichment [25].

### Validation of DEGs using qRT-PCR

Real-time quantitative reverse transcription PCR (RT-PCR) was performed at 37°C for 15 min followed by 85°C for 5 s by using the PrimeScript RT Master Mix Kit (Takara, USA). The reactions of 20 μl were prepared using 10 μl SYBR Premix Ex Taq II, 0.8 μl of 10 mM forward and reverse primers each, sterile water and 1 μl cDNA template following which amplification reactions were conducted. The cycling parameters used were; 95°C for 30 s, 40 cycles of 95°C for 5 s and 60°C for 30 s [26]. All reactions were carried out in triplicate, and the data obtained were analyzed using the ABI 7500 Software v2.3, and Livak's -ΔΔ CT method was used for gene expression data analysis [27]. The PCR primer pairs used in qRT-PCR are shown in Table 1.

**Table 1:** Primer sequences of genes used for validation study using qRT-PCR.

S. No.	Chro. No.	Gene ID	Forward Primer	Reverse primer	Amplicon length (bp)	Gene Name / Functional description from Cotton FGD* [28]
1	A05	Gh_A05G3682	GTTGGGGCTTCGAAGGGTAA	AACGTTGCTTGAGGAGTGGT	133	Beta-galactosidase 16
2	D11	Gh_D11G3302	AAGATCCCCGGCGTTTACTC	ATGGGGGACTTGGGAGTGTA	118	Expansin A4
3	D04	Gh_D04G1783	AAAGGCTACGGCAATGGACA	TGCCTCCACCTTACATGCAG	129	Pectin lyase-like superfamily protein
4	A01	Gh_A01G0900	GGATGGCATCGACTTTTGTGG	AATGCAAAAACGCCTTGCGA	105	Tubulin beta-1 chain
5	D07	Gh_D07G0486	GTTGCCCTAGTGAGTGCGAT	ATCCGAGCTTTCTTGGGAGC	149	Homolog of Medicago truncatula MTN3
6	A08	Gh_A08G0788	TCGATTTCTACAGTACCAGCAG	TTATGACGGCTGCCCTTCTC	109	Seven transmembrane MLO family protein
7	A01	Gh_A01G1209	CCAAATGATTGGCCTTGCCC	CACCACCCTCATTGTGACCA	103	TBP-associated factor 15
8	D11	Gh_D11G3072	CCCGAGAACACTACCAACGAC	CGGTTTCCCTTCAACAGCCT	150	Acyl-CoA-binding protein 6
9	A05	Gh_A05G0419	GGCAATGGCCATCTTCACAC	ACTAGGCCCGTCTTCATCCT	145	Reticulon family protein
10	D01	Gh_D01G1631	CCAGCAAACCCAACCAGAGT	AGTAGTGATGGTGCTGCAAGT	109	myb domain protein 4
11	A04	Gh_A04G1346	AAAACGCACGTCACCTACCT	TCCGGTACTGTTTGCTTCGG	10	DUF828 Plant protein with unknown function
12	A11	Gh_A11G0133	ATCCCTCCTCTCGTCGGAAA	GGTTGCCCGCATTGTTCTTT	111	RNA-binding family protein
13	A01	Gh_A01G0153	CCGGAGCTTCCGAGGTATTC	CTCGACGGAACCGAAAAGGA	129	Jasmonate-zim-domain protein 10
14	A09	Gh_A09G0200	AAGACGTTCTCACGGCCATT	TTCCTCCATCCAGCAAAGCC	144	Inositol monophosphatase family protein
15	D04	Internal Control	GCGATCTGGTAAGGAGCTTG	GGAGAAGGTTTCCACAACCA	106	*Gossypium hirsutum* EF1A1

* Cotton Functional Genomics Database (CottonFGD) is a validated integrates transcriptomic database for analysis platform for cotton (*Gossypium* spp).

An overview of the implemented workflow to identifying DEGs, GO, SEA, and KEGG pathway analysis of three different lines of cotton (*Gossypium hirsutum* L.) is given in (Fig 1).

## Results

### Transcriptome sequencing and assembly

The present study was resulted to identify the DEGs involved in CMS pathways by comparing the CGMS, maintainer and restorer lines of cotton (*Gossypium hirsutum* L.). The cDNA libraries for three lines i.e. JS178, JB178 and JR178 during SS and MS stages were prepared separately, and reads were pooled for both stages of each line accounting 53.05, 61.70 and 67.96 million raw reads, respectively. The result of quality trimming as well as mapping against cotton reference genome using the STAR version 2.5.1 is given in Table 2.

**Table 2:** Quality screening and mapping statistics of RNA-Seq data.

Reads	JS178 (CGMS)	JB178 (Maintainer)	JR178 (Restorer)
**No. of reads generated**	53,048,502	61,704,635	67,960,025
**No. of reads passed quality trimming**	48,457,321	55,645,046	61,579,735
**No. of reads mapped to reference genome of Cotton (*Gossypium hirsutum* L.)**	34,037,349 (70.24%)	39,800,204 (71.52%)	45,395,238 (73.71%)
**Total no. of bases mapped**	5,325,913,900	6,227,644,626	7,103,116,022
**GC% of mapped reads**	43.17	43.24	43.26
**Q25% of mapped reads**	94.23	94.19	94.26
**Error% of mapped reads**	0.0128	0.0114	0.0112
**Average length of mapped reads (bp)**	237	234	245

### SEA, GO and KEGG classification of the transcriptome

**Fig 2:**
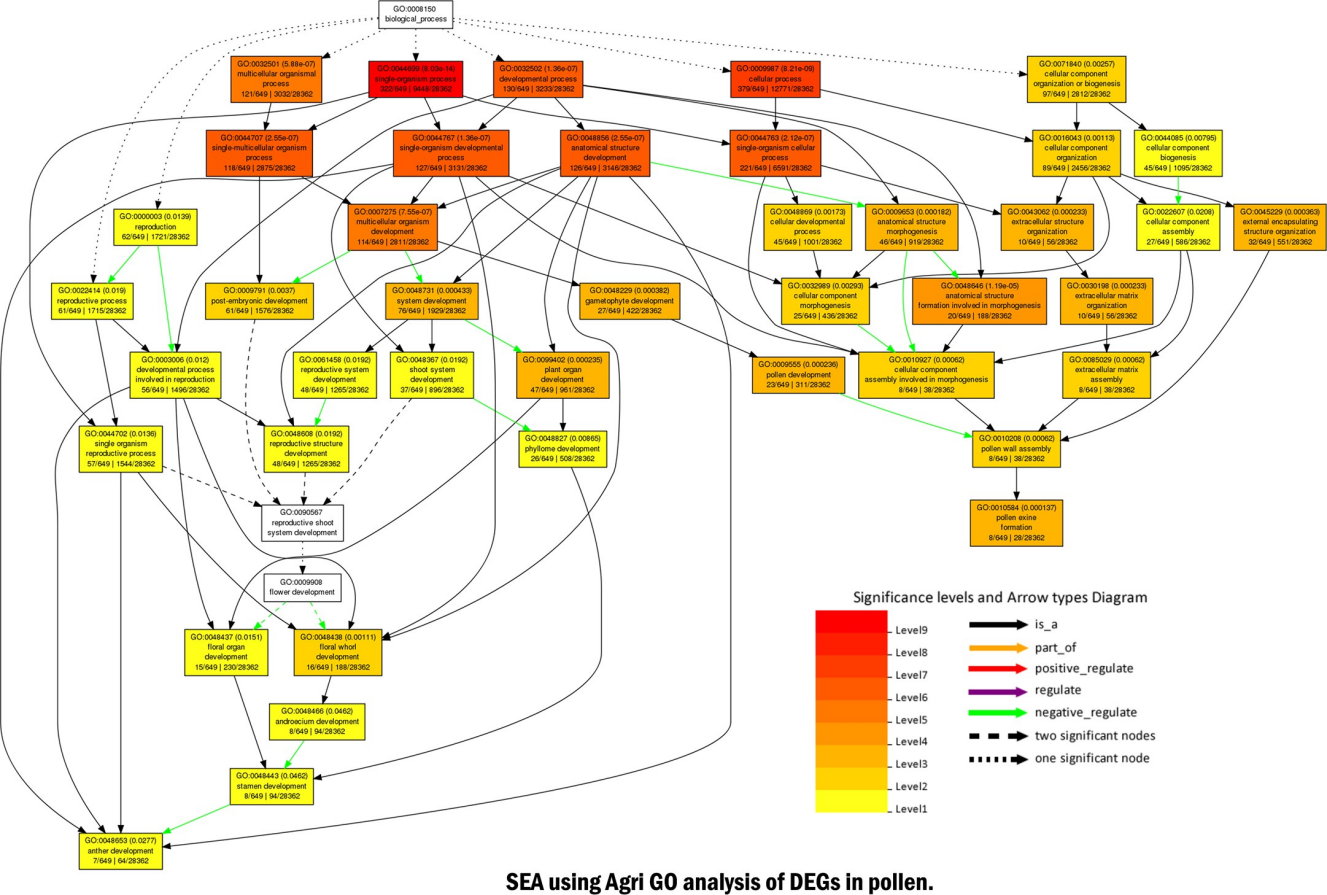
Agri GO analysis of DEGs in floral buds. Singular enrichment analysis was performed in AgriGO v2 to identify enriched gene ontologies associated with pollen developmental processes. Each box shows the GO term number, the p-value in parenthesis, and GO term. The pair of numerals in the left represents the number of genes in input list associated with that GO term and number of genes in the input list. The pair of numerals in the right represents the number of genes associated with a targeted GO term in the Gossypium database and a total number of Gossypium genes with GO annotations in the Gossypium database. Colour and design of arrow indicate the effect of pathways. Box colors indicate levels of statistical significance: yellow  =  0.05; orange  =  e−5; and red  =  e−9.

**Fig 3:**
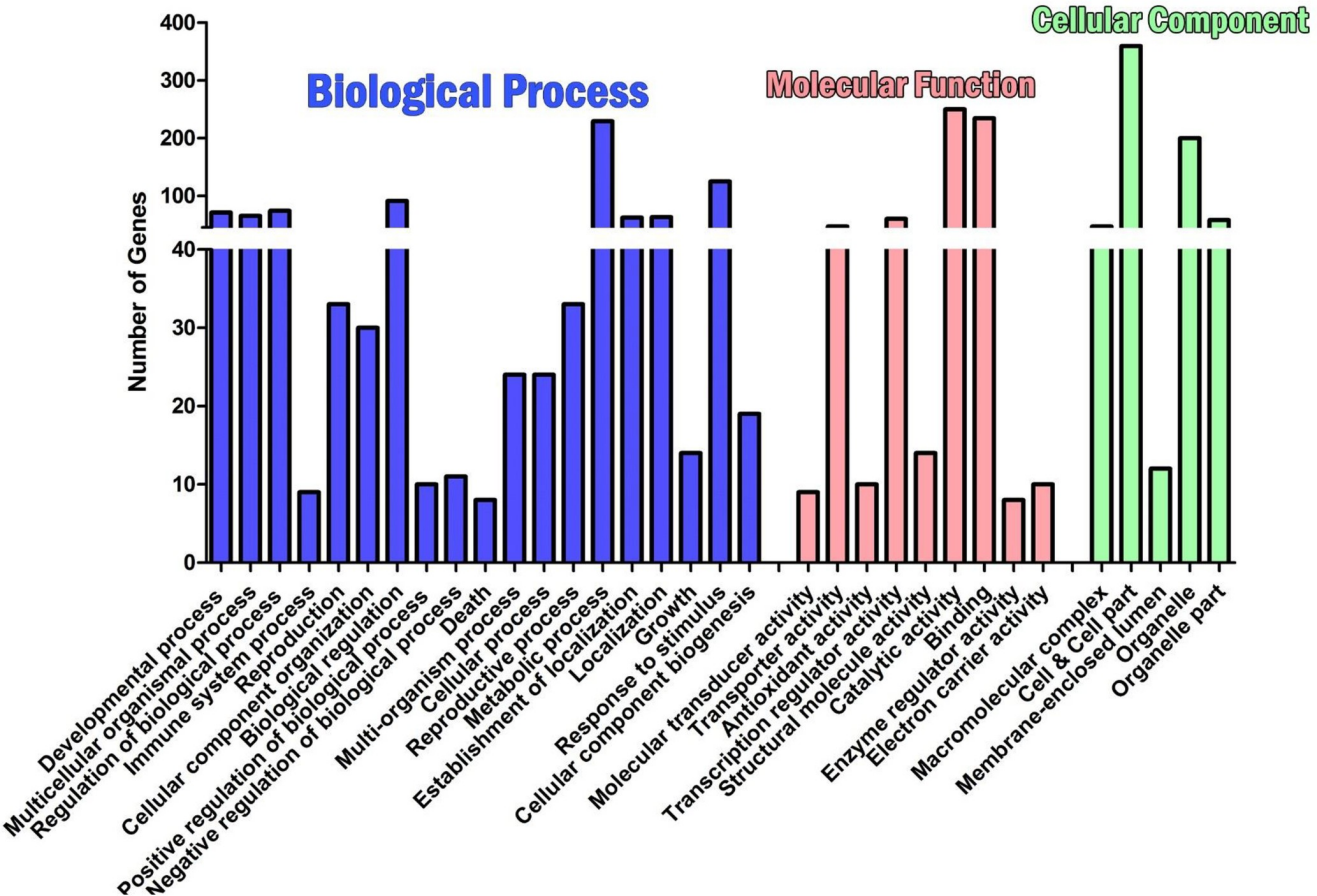
GO classification of non-redundantly expressed genes in CGMS, maintainer and restorer lines of *Gossypium hirsutum* L.

AgriGo v2 was utilized to perform SEA to identify GO terms and associated pathways analysis revealed that, the anther development is negatively regulated via stamen, androecium, floral whorl & organ developments at a different level with negative regulation of pathways related to pollen wall assembly and pollen exine formation. The details of each pathway with a level of their involvement is given in (Fig 2). A total of 2,313 predicted transcripts were assigned GO annotations in three main GO categories and 32 subcategories (Fig 3). The Biological Process category of GO is mainly enriched with ‘Metabolic process’ (229 genes), ‘Response to stimulus’ (129 genes) and ‘Biological regulation’ (91 genes) representing 23.01%. 12.56% and 10% of total transcript involved in this category. The ‘Cell and Cell part’ (359 genes; 52.66%), ‘Organelle’ (200 genes; 29.5%) and ‘Organelle part’ (58 genes; 8.5%) had the most number of genes in the cellular component category. The molecular function category of GO was mainly sub-categorized by ‘Catalytic activity’ (250 genes; representing 38 .9% of transcripts in the category), ‘binding’ (234 genes; 36.4%) and ‘Transcription regulator activity’ (60 genes; 10%) (S1 Table). In addition, the specific biological functions of DEGs and their metabolism networks were analyzed using KEGG pathway analysis, and a total of 2201 transcripts were classified into 110 pathways (S2 Table), among which metabolic pathways, biogenesis of secondary metabolites and signal transduction in plant hormones contained the most transcripts.

### DEGs between sterile and fertile buds

**Fig 4:**
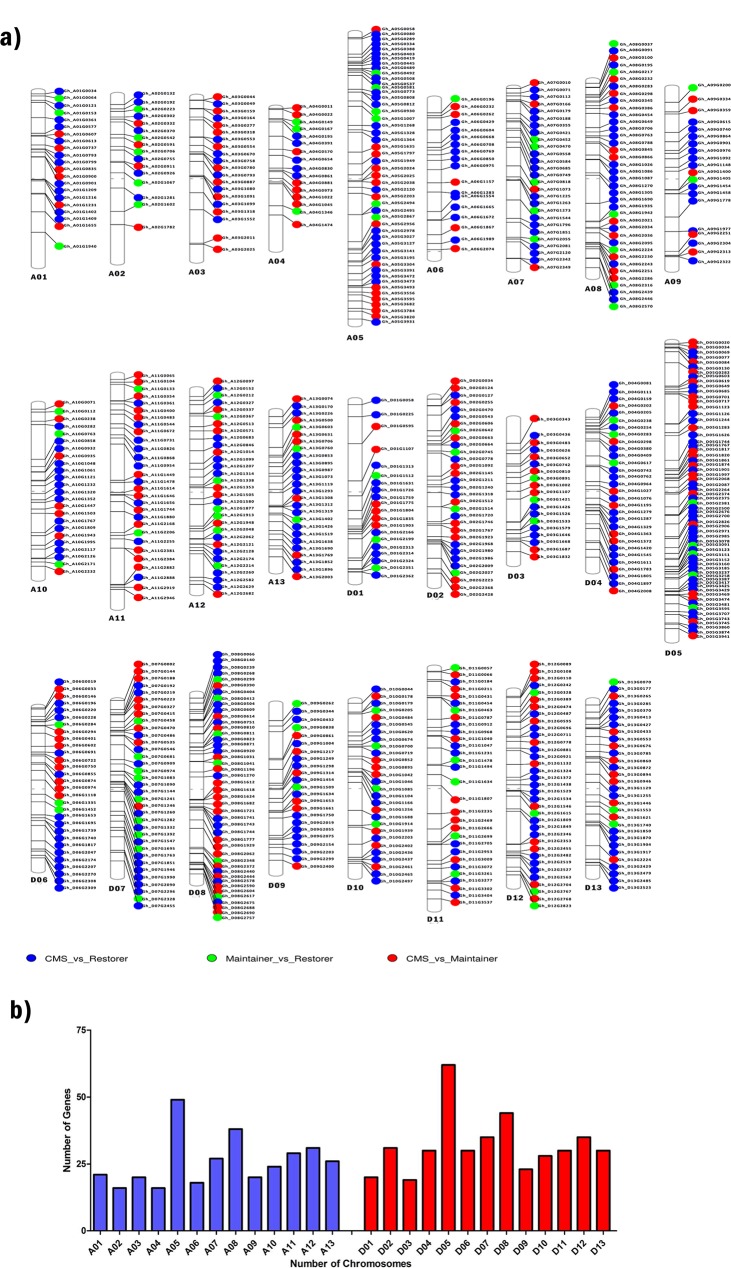
**a) Distribution of differentially expressed genes on chromosomes of *Gossypium hirsutum* L**. Distribution of phenogram showing the exact location of DEGs on each chromosome of *Gossypium hirsutum* L. **b) The total number of DEGs present on each chromosome of cotton**. A total number of differentially expressed genes on diploid sets of Gossypium genome A and D. The X-axis represents different chromosomes. Y-axis is DEGS numbers on each chromosome.

A total of 754 non-redundant significantly DEGs between JS178, JB178, and JR178 were identified using Cufflinks. The exact location of the DEGs was identified and plotted on 26 chromosomes of G. *hirsutum* using Phenogram software (Fig 4A).

The total number of DEGs present on each chromosome were presented in (Fig 4B). Location of DEGs on cotton chromosomes showed a great bias towards parenteral genome of *G*. *raimondii* genome (D) as compared to its counterpart. Moreover, it was found that chromosome number 5 has the highest number of DEGs followed by 8 and 12.

**Fig 5:**
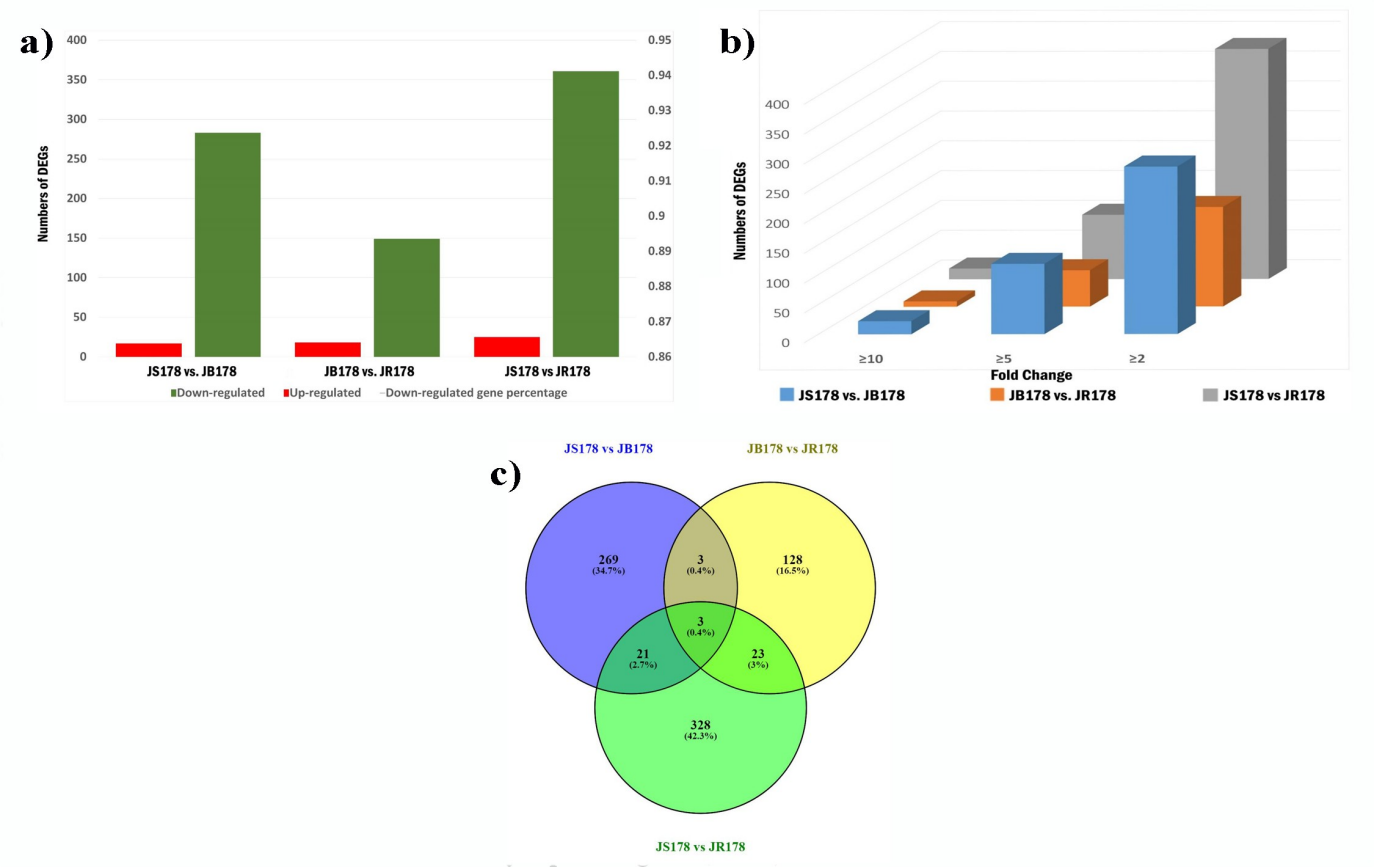
**a) The number of differentially expressed genes (DEGs) between three different lines of cotton.** The green color represented up-regulated, the red color represented down-regulated gene numbers on the X-axis, Y-axis represented the number of deferentially expressed genes. **b) The fold change wise distributed numbers of genes under the comparison of three different lines. c) Venn diagram showing the distribution of unique and common DEGs among three comparisons**.

The comparison of three lines among each other revealed, 17 up-regulated and 283 down-regulated DEGs in JS178 compared to JB178 (Fig 5A; S3 Table); 19 up-regulated and 149 down-regulated in JB178 compare to JR178 (Fig 5A, S4 Table); and 28 up-regulated and 361 down-regulated in JS178 compared to JR178 (Fig 5A; S5 Table).

A total of 300 DEGs are found by comparing JS178 and JB178, which are assumed to have cytoplasmic differences. These DEGs includes, 11 protein-coding genes (S6 Table), possibly targeted to the mitochondria, which were highly down-regulated in JS178 in addition to 3 chloroplast targeted protein-coding genes Gh_A07G0166, Gh_D07G0223 and Gh_A09G1400. The same set of genes are having a different level of expression when the comparison was made between JS178 and JR178, which are likely to have differences in both nuclear and cytoplasmic genome. The current study found only two common genes, of which Gh_D07G1246 has similar fold change in both compressions and another one i.e. Gh_A08G0763 was more down-regulated in JS178 compare to JR178. This DEGs may be affected by the interaction of the CMS cytoplasm and male sterile nucleus genes so can be a good candidate for more study on CGMS in cotton.

The total number of DEGs between the three lines in terms of various fold change is presented in Fig 5B. Genes with >10 fold differences, accounted for 82.62%, of all non-redundant DEGs. These genes mainly encoded for transcription factor myb domain protein 4, *AMS*, *DYT1*, bHLH DNA-binding family protein, WD40 repeat like superfamily protein, VDAC4_ARATH mitochondrial outer membrane protein porin, and NAD (P)-binding superfamily protein. The results indicated that 21 shared DEGs with the similar expression pattern were identified in both between JS178 vs. JR178, as well as JS178 vs. JB178 (Fig 5C). All these common genes displayed high down-regulation in the CGMS line, mainly includes, 6 transcription factors; 3 cytochrome P450 family; 2 each of xylem bark cysteine peptidase and chaperone DnaJ-domain superfamily protein; 6 protein of DUF581 with unknown function; 4 tubulin alpha-2 chain; one each of ribosomal protein L39 family protein, protein kinase gene, ubiquitin-conjugating enzyme 34, ABHD protein, pectin lyase like superfamily protein and HAD superfamily.

Transcription factors (TFs) play a vital part in various biological processes by regulating gene transcription by binding to specific DNA sequences in the regulatory regions of multiple target genes [29]. Therefore, even a minor alteration in the expression of TFs normally leads to intense changes in the gene expression level. Our analysis identified 12 up-regulated (like Homeodomain-like superfamily protein, Chaperone DnaJ-domain superfamily protein, acyl-CoA -binding protein 6, multiprotein bridging factor 1A, MADS-box TF family protein and AGAMOUS-like 19) and 63 (including TFs belong to bHLH, NAC, WRKY transcription factor family, Heat shock protein, *MYB*, F-Box, bZIP transcription factor family protein and GATA-type ZFTF family protein) (S7 Table).

Pathway-based analysis of the deregulated genes were done using KEGG automatics annotation server and classified utilizing the single-directional best hit strategy [30]. In JS178 compared with the fertile lines (JB178 and JR178), most of the up-regulated DEGs functioned as ‘ribosome’ (S8 Table). In contrast, the large number of down- regulated DEGs functioned as ‘metabolic pathways’, ‘biosynthesis of secondary metabolites, pentose and glucuronate inter-conversions’, carbon metabolism, plant-pathogen interaction, and plant hormone signal transduction (S9 and S10 Tables).

GO analysis showed that, the up-regulated DEGs in the sterile line mainly regulates the metabolism of organonitrogen compounds, nucleobase-containing small molecule metabolic process and non-membrane-bounded organelle. While, down-regulated DEGs mainly functioned in the single-organism processes, anther wall tapetum development process, pollen development, carbohydrate metabolism and cell wall biogenesis or organization. For molecular function, main GO terms of DEGs contained tetrapyrrole binding, oxidoreductase activity, hydrolase activity, acting on ester bonds. For cellular component, five GO terms of DEGs were accessible, which participated in the extracellular region, apoplast and COPII vesicle coat (S11 Table).

### DEGs involved in cell wall component

In current study, all the 28 genes down-regulated in sterile line were associated with anther wall tapetum, pollen exine and cell wall formation. These genes may be related to the abortive phenotypes anther and pollen development and consequent resultant sterile lines. Gh_D12G2768, Gh_A11G2384, and Gh_D11G0403 are highly down-regulated by 12.06, 12.91 and 13.34 folds respectively. These are important genes involved in degradation of callose and functional regulation of developmental stages of tapetum. In addition to tapetum related genes, 23 genes related to cell wall components were identified including acyl-CoA synthetase 5, 4 cellulose synthase, 2 CYP450 family polypeptide, 6 expansin (A4 and A8), 4 xyloglucan hydrolase, 2 pectin methylesterase inhibitor superfamily, Pectinacetylesterase family protein, Nucleotide-diphossugar_trans and, myb domain protein 4. All these genes were down-regulated in JS178. These genes contribute to the alteration of intine formation in JS178.

### Differential expression of Pentatricopeptide Repeat (PPR) Proteins and carbohydrate metabolism related activity

Genes encoding the site-specific PPR motif are arbitrating through the interactions between RNA-binding substrates and enzymes. It play a critical role in the biogenesis of plant mitochondria [31]. It was observed that genes encoding PPR proteins i.e. Gh_D05G2657, Gh_A01G0058, Gh_D05G0682, Gh_D05G0343 and Gh_D08G0123 were down-regulated in JS178 compared to the restorer line. Besides these genes, 2 PPR genes Gh_D12G0614 and Gh_D12G0539 were highly down-regulated in maintainer when compared to restorer line JR178. These PPR proteins could be candidates for examining the co-operation between mitochondrial and nuclear gene expression [32].

Rearrangements in the mitochondrial genome can modify the expression profile of mitochondrial genes or cause reduction in carbohydrate accumulation [33]. Oxidative stress could lead to premature abortion of tapetal cells [34]. In the present study, 43 genes involved in carbohydrate metabolic and 58 involved in oxidoreductase activity were differentially expressed between sterile and fertile lines.

### Concordance check of RNA-Seq profile using qRT-PCR

**Fig 6:**
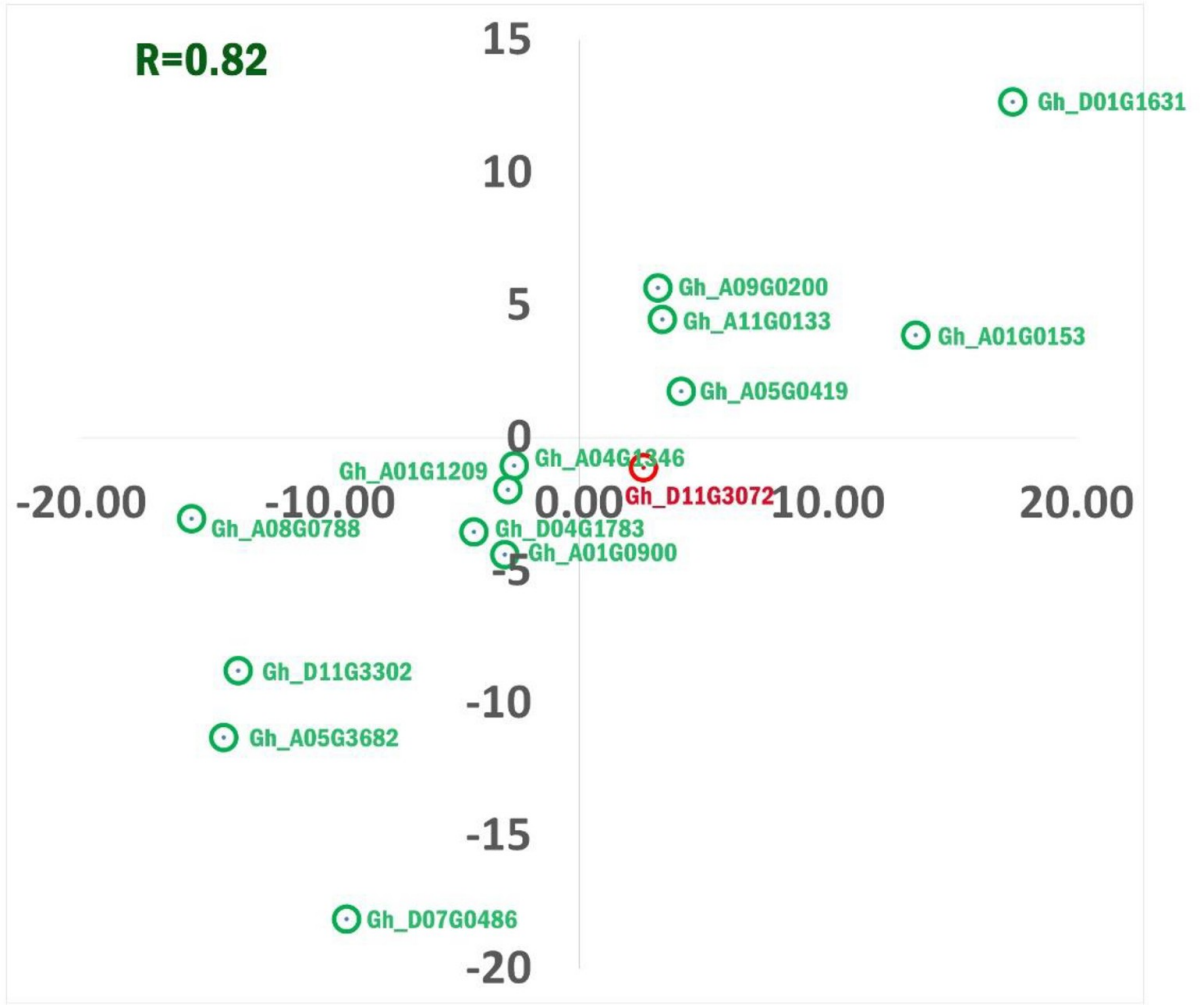
qRT-PCR analysis of gene expression compared with the RNA-seq data. Expression of 14 transcript as analyzed by quantitative PCR in CGMS and fertile lines of *Gossypium hirsutum* L. The dots with green colour (thirteen in numbers) indicates concordance whereas, red (one in number) indicates non-concordance of expression profiling in qRT-PCR and RNA-seq.

To evaluate the results of RNA-Seq with concordance of patterns of DEGs, we have investigated 14 DEGs (8 down and 6 up-regulated) by qRT-PCR assays from the same RNA samples that were used for the transcriptome analysis [35]. The corresponding primers use in this study are listed in Table 2. Out of the 14 transcripts subjected to validation in 3 different samples, thirteen transcripts showed complete concordance with the expression profile of RNA-Seq analysis and 1 transcript namely, Gh_D11G3072, showed contrasting expression profile. There was apparently a strong positive correlation (R^2^ = 0.82) between the data obtained from RNA-Seq and qRT-PCR data. Dissimilarity between the biological replications was non-significant in real time PCR. Thus, based on these results, we confirmed that RNA-Seq was highly consistent with qRT-PCR. The correlation of RNA-Seq profile and qRT-PCR result for each gene tested is given in Fig 6 (S12 Table).

## Discussion

In the current study, a relative transcriptome investigation of anthers of three lines of cotton was conducted to recognize molecular portraits that are differentially communicated in the fertile and sterile cotton plants for encouraging the distinguishing proof of systemic gene expression, regulatory pathways and mechanisms involved with CMS in cotton. The most of the DEGs in JS168 (94.33% vs JB178; and 93.52% vs JR178, Fig 4A) were down-regulated compared to fertile lines. A similar comparative pattern was also detailed in transcriptome study between CMS of *Gossypium harknessii*, its maintainer and restorer lines [12]. In another investigation to distinguish genes contributing towards faulty pollen wall in male sterility, more number of down-regulated genes were recognized in sterile line [36, 37]. Total number of DEGs identified between JB178 and JR178 were less than other comparisons indicating regulatory roles of restorer genes in addition to the role of reproductive rehabilitation [38]. Most of DEGs were located in genome D especially Chr_D05. This result is similar to the study of Wu, Zhang (9) and Zhao [39] where they distinguished 20 hopeful genes including *Rf1*, for fertility restoration on chromosome 5 in cotton.

The fruitful collaboration of gametophytic and sporophytic genes results in the propagation of flowering plants through the development of viable pollen grains of the anther [40, 41]. The link between these two procedures occurs in tapetum. The secretory tissue of the tapetum plays an important role by giving basic essential nutritional components required for the development of pollen and exine formation [42]. In the present investigation, aberrant expression of genes required for anther and pollen cell wall development were seen at various levels. The development of pollen exine mainly depends on the tapetum, which begins depositing lipidic components of tryphine into exine cavities which results in the formation of the sculptured exine [43]. The synergy between tapetal PCD and microspore development is inevitable. Several TFs involved in guiding the development of the tapetum and pollen arrangement have been reported [44]. In this study, six MYB genes (Gh_A05G2120, Gh_D07G2090, Gh_D06G0691, Gh_A09G1458, Gh_D01G1631 and Gh_A07G2342) were observed to be down-regulated in sterile lines. AtMYB103 particularly expressed in tapetum and in the middle layers of anthers assumes to have an essential role in the development of the tapetum and pollen, callose disintegration, exine arrangement in anthers. Reduction in the expression of AtMYB103 resulted in an earlier degeneration of tapetum, and majority of pollen grains lacked cytoplasm and were metamorphopsised [45, 46]. Additionally, bHLH which is noted as tapetum-particular transcription factors were reported by many researchers to play critical role in tapetum and pollen developmental process [47–50], stomata development and hormone signaling [51]. In the current study, six bHLH genes (Gh_A07G0112, Gh_D10G0179, Gh_A11G1614, Gh_A12G0337, Gh_D06G1739 and Gh_D06G1740) were found to be down-regulated in sterile lines. One of the most interesting down-regulated bHLH in sterile line (-14.05 fold) Gh_D10G0179, corresponding to AT4G21330 which acts as a DYT1_ARATH transcription factor dysfunctional tapetum. Arabidopsis dysfunctional tapetum 1 (DYT1), was the first identified bHLH transcription factor which specifically modulates the function and development of the tapetum via regulating thousands of anther genes. A total of 276 genes have been reported to be deregulated through the feed- forward regulatory loops between the downstream bHLH transcription factors, other bHLH gene families and *DYT1* [47]. The one more promising target among bHLH gene i.e. Gh_A12G0337, was down-regulated in sterile line by more than 13 fold. It encodes for ‘Aborted Microspores (AMS)’ gene, which is key regulator of pollen wall formation [52]. The *AMS* encodes many of bHLH family protein. The mutant of *AMS* shows deterioration of tapetum as well as microspores at anther stage 7 [53].

The current study also traced many distinguishably down-regulated genes in the floral buds of JS178 which were related to pollen cell wall formation, pollen development, and pollen tube growth. These genes include Expansin, Cytochrome P450, and COBRA-like gene. In maize, expansin expedites pollen tube growth through breaking up the maternal cell walls [54]. COBRA-Like genes (*COBLs*) are mainly responsible for the secondary cell wall biogenesis [55]. The mutations COBl4 in *A*. *thaliana* were accounted for to influence the mechanical quality of vascular bundles and have a huge decrease in cellulose content [56]. P450 family are involved in anther pollen development [57]. Li et al 2010 announced that CYP704B2 a family member of Cytochrome P450 is necessary for cuticle and exine formation during plant male gamete formation [58].

Fertility rebuilding for CMS crops is the most essential concern in heterosis utilization and the creation of hybrid seeds [59]. The hereditary studies demonstrated that PPRs tie to a particular regulatory sequence of RNA and develop anterograde regulation like, post-transcriptional splicing, editing, processing, or regulating mRNA stability [60, 61]. In this study, 1 up-regulated and 7 down-regulated PPR genes (Gh_D05G2657, Gh_A01G0058, Gh_D05G0682, Gh_D05G0343, Gh_D08G0123, Gh_D12G0614 and Gh_D12G0539) were identified in the sterile line. The hindering impact of CMS proteins on the development of the tapetum and pollen can be prevented by the interaction between members of CMS and Rf family members through various mechanisms [62]. About half of the identified Rf genes encode Pentatricopeptide Repeat **(**PPR**)** [63, 64]. Emp5 gene is a corn PPR gene that regulates mitochondrial gene expression through altering transcripts of the ribosomal L16 rpl16- 458 locus, prompts modification in seed advancement [65]. Chen and Liu (62) reported that PPR genes by hindering the expression of mitochondrial CMS genes could re-establish pollen fertility in many plant species [62]. Therefore, we could hypothesize that the JS178 genes and pathways that regulated male sterility are not only associated with the mitochondria but are also involved in nuclear–mitochondria interaction.

The current study also found 5 down-regulated F-box TFs in the JS178 line. Li, Li [66] demonstrated that suppression of F-box protein-encoding gene, OsADF (anther development F-box), causes degeneration of tapetum and finally a male sterility in rice. WRKY-family (Gh_D07G2328, Gh_A11G0868, Gh_A11G0954, and Gh_A12G2121) and bZIP TFs family protein (Gh_A01G1655, Gh_A09G0901, and Gh_A06G1283) were additional TFs, which were highly down-regulated in JS178. The WRKY-family and bZIP transcription factors have an established role in pollen and anther development pathways [67].

GO enrichment analysis of 55 DEGs located on chromosome D05, showed enriched pathways related to catalytic activity, carbohydrate metabolic process and reproduction possibly suggesting pathways of deprivation of soluble sugar could be a cause male sterility as observed by another group of researchers [39, 68].

## Conclusions

The reconnaissance of male-sterility associated genes and the clear understanding of the functions of these genes in cotton has high value in it’s breeding programmes. In the present study, the examination of the whole transcriptome of CGMS, maintainer and sterile lines of *Gossypium hirsutum* L. resulted in the identification of 754 non-redundant DEGs consist of 53 up and 701 down-regulated DEGs in the sterile line as compare to fertile lines. The signaling pathway investigation through GO and KEGG enrichment analysis showed carbohydrate metabolism-related genes, cellular activity related genes like cell division, cell wall formation and development, cell membrane, and cytoskeleton as well as transcription factors which have a role in development of reproductive organs such as pollen wall formation, pollen maturation, pollen tube and anther development were far-reaching pathways altering among fertile and sterile lines. These data validated the already uncovered transcript related to sterility pathways in other plant species also exist in cotton. It is notable that in sterile line highly down-regulated genes *DYT1* and *AMS*, which are involved in tapetum function, anther, and pollen development might be appropriate candidate genes for functional analysis of gene pertaining the genetic lattice that controlled the functional development of anther and pollen. The present investigation provide useful data for future investigations to uncover the biology behind the cyto-nucleoplsmic interaction leading sterility and restoration of in cotton.

## Supporting information

S1 Table GO classification of expressed genes in CGMS, maintainer and restorer lines of *Gossypium hirsutum* L. (XLS)

S2 Table KEGG Pathway analysis of all expressed genes in CGMS, maintainer and restorer lines of *Gossypium hirsutum* L. (XLS)

S3 Table A detailed information of expression profile of DEGs between JS178 and JB178. (XLS)

S4 Table A detailed information of expression profile of DEGs between JB178 and JR178. (XLS)

S5 Table A detailed information of expression profile of DEGs between JS178 and JR178. (XLS)

S6 Table List of mitochondrial genes down-regulated in flower buds of JS178 line compare to fertile lines. (XLS)

S7 Table A detailed of differentially expressed transcription factor between sterile and fertile lines. (XLS)

S8 Table KEGG analysis of the up-regulated DEGs between sterile and fertile lines. (XLS)

S9 Table KEGG analysis of down-regulated DEGs of JS178 vs JB178. (XLS)

S10 Table KEGG analysis of down-regulated DEGs of JS178 vs JR178. (XLS)

S11 Table GO classification of the down-regulated DEGs between sterile and fertile lines. (XLS)

S12 Table List of primers with details of sequence and expression profile used for qRT-PCR validation (14 randomly selected transcripts). (XLS)

## References

[1] Salih H, Gong W, He S, Mustafa NS, Du X. Comparative transcriptome analysis of TUCPs in Gossypium hirsutum Ligon-lintless-1 mutant and their proposed functions in cotton fiber development. Molecular Genetics and Genomics. 2018:1–12. 10.1007/s00438-018-1482-x 30159616

[2] Park Y-H, Alabady MS, Ulloa M, Sickler B, Wilkins TA, Yu J, et al Genetic mapping of new cotton fiber loci using EST-derived microsatellites in an interspecific recombinant inbred line cotton population. Molecular Genetics and Genomics. 2005;274(4):428–41. 10.1007/s00438-005-0037-0 16187061

[3] Yu S, Fan S, Wang H, Wei H, Pang C. Progresses in research on cotton high yield breeding in China. Sci Agric Sin. 2016;49:3465–76.

[4] Fang W, Zhao Fa, Sun Y, Xie D, Sun L, Xu Z, et al Transcriptomic profiling reveals complex molecular regulation in cotton genic male sterile mutant Yu98-8A. PLoS One. 2015;10(9):e0133425 10.1371/journal.pone.0133425 26382878 PMC4575049

[5] Longin CFH, Gowda M, Mühleisen J, Ebmeyer E, Kazman E, Schachschneider R, et al Hybrid wheat: quantitative genetic parameters and consequences for the design of breeding programs. Theoretical and applied genetics. 2013;126(11):2791–801. 10.1007/s00122-013-2172-z 23913277

[6] Nie H, Wang Y, Su Y, Hua J. Exploration of miRNAs and target genes of cytoplasmic male sterility line in cotton during flower bud development. Functional & integrative genomics. 2018:1–20. 10.1007/s10142-018-0606-z 29626311

[7] Pruitt KD, Hanson MR. Transcription of the Petunia mitochondrial CMS-associated Pcf locus in male sterile and fertility-restored lines. Molecular and General Genetics MGG. 1991;227(3):348–55. 1865874 10.1007/BF00273922

[8] Chen Z, Zhao N, Li S, Grover CE, Nie H, Wendel JF, et al Plant mitochondrial genome evolution and cytoplasmic male sterility. Crit Rev Plant Sci. 2017;36(1):55–69.

[9] Wu J, Zhang M, Zhang B, Zhang X, Guo L, Qi T, et al Genome-wide comparative transcriptome analysis of CMS-D2 and its maintainer and restorer lines in upland cotton. BMC genomics. 2017;18(1):454 10.1186/s12864-017-3841-0 28595569 PMC5465541

[10] Li F, Fan G, Lu C, Xiao G, Zou C, Kohel RJ, et al Genome sequence of cultivated Upland cotton (Gossypium hirsutum TM-1) provides insights into genome evolution. Nature biotechnology. 2015;33(5):524 10.1038/nbt.3208 25893780

[11] Wang S, Wang C, Zhang X-X, Chen X, Liu J-J, Jia X-F, et al Transcriptome de novo assembly and analysis of differentially expressed genes related to cytoplasmic male sterility in cabbage. Plant physiology and Biochemistry. 2016;105:224–32. 10.1016/j.plaphy.2016.04.027 27116370

[12] Han Z, Qin Y, Deng Y, Kong F, Wang Z, Shen G, et al Expression profiles of a cytoplasmic male sterile line of Gossypium harknessii and its fertility restorer and maintainer lines revealed by RNA-Seq. Plant Physiology and Biochemistry. 2017;116:106–15. 10.1016/j.plaphy.2017.04.018 28551417

[13] Liu Q, Lan Y, Wen C, Zhao H, Wang J, Wang Y. Transcriptome sequencing analyses between the cytoplasmic male sterile line and its maintainer line in welsh onion (Allium fistulosum L.). International journal of molecular sciences. 2016;17(7):1058. 10.3390/ijms17071058 PMC4964434 27376286

[14] Chen P, Ran S, Li R, Huang Z, Qian J, Yu M, et al Transcriptome de novo assembly and differentially expressed genes related to cytoplasmic male sterility in kenaf (Hibiscus cannabinus L.). Molecular breeding. 2014;34(4):1879–91.

[15] Qiu Y, Liao L, Jin X, Mao D, Liu R. Analysis of the meiotic transcriptome reveals the genes related to the regulation of pollen abortion in cytoplasmic male-sterile pepper (Capsicum annuum L.). Gene. 2018;641:8–17. 10.1016/j.gene.2017.10.022 29031775

[16] Omidvar V, Mohorianu I, Dalmay T, Zheng Y, Fei Z, Pucci A, et al Transcriptional regulation of male-sterility in 7B-1 male-sterile tomato mutant. PLoS One. 2017;12(2):e0170715 10.1371/journal.pone.0170715 28178307 PMC5298235

[17] Hamid R, Tomar RS, Marashi H, Shafaroudi SM, Golakiya BA, Mohsenpour M. Transcriptome profiling and cataloging differential gene expression in floral buds of fertile and sterile lines of cotton (Gossypium hirsutum L.). Gene. 2018;660:80–91. 10.1016/j.gene.2018.03.070 . 29577977

[18] Yang P, Han J, Huang J. Transcriptome sequencing and de novo analysis of cytoplasmic male sterility and maintenance in JA-CMS cotton. PloS one. 2014;9(11):e112320 10.1371/journal.pone.0112320 25372034 PMC4221291

[19] Yingru W, Llewellyn DJ, Dennis ES. A quick and easy method for isolating good-quality RNA from cotton (Gossypium hirsutum L.) tissues. Plant Molecular Biology Reporter. 2002;20(3):213–8. 10.1007/BF02782456

[20] Dobin A, Davis CA, Schlesinger F, Drenkow J, Zaleski C, Jha S, et al STAR: ultrafast universal RNA-seq aligner. Bioinformatics. 2013;29(1):15–21. 10.1093/bioinformatics/bts635 23104886 PMC3530905

[21] Trapnell C, Roberts A, Goff L, Pertea G, Kim D, Kelley DR, et al Differential gene and transcript expression analysis of RNA-seq experiments with TopHat and Cufflinks. Nature protocols. 2012;7(3):562 10.1038/nprot.2012.016 22383036 PMC3334321

[22] Li J, Han S, Ding X, He T, Dai J, Yang S, et al Comparative transcriptome analysis between the cytoplasmic male sterile line NJCMS1A and its maintainer NJCMS1B in soybean (Glycine max (L.) Merr.). PLoS One. 2015;10(5):e0126771 10.1371/journal.pone.0126771 25985300 PMC4436259

[23] Wolfe D, Dudek S, Ritchie MD, Pendergrass SA. Visualizing genomic information across chromosomes with PhenoGram. BioData mining. 2013;6(1):18 10.1186/1756-0381-6-18 24131735 PMC4015356

[24] Tian T, Liu Y, Yan H, You Q, Yi X, Du Z, et al agriGO v2. 0: a GO analysis toolkit for the agricultural community, 2017 update. Nucleic acids research. 2017;45(W1):W122–W9. 10.1093/nar/gkx382 28472432 PMC5793732

[25] Xie Y, Zhang W, Wang Y, Xu L, Zhu X, Muleke EM, et al Comprehensive transcriptome-based characterization of differentially expressed genes involved in microsporogenesis of radish CMS line and its maintainer. Funct Integr Genomics. 2016;16(5):529–43. 10.1007/s10142-016-0504-1 27465294

[26] Koringa PG, Jakhesara SJ, Bhatt VD, Meshram CP, Patel AK, Fefar DT, et al Comprehensive transcriptome profiling of squamous cell carcinoma of horn in Bos indicus. Veterinary and comparative oncology. 2016;14(2):122–36. 10.1111/vco.12079 . 24314272

[27] Livak KJ, Schmittgen TD. Analysis of relative gene expression data using real-time quantitative PCR and the 2− ΔΔCT method. Methods. 2001;25(4):402–8. 10.1006/meth.2001.1262 11846609

[28] Zhu T, Liang C, Meng Z, Sun G, Meng Z, Guo S, et al CottonFGD: an integrated functional genomics database for cotton. BMC plant biology. 2017;17(1):101 10.1186/s12870-017-1039-x 28595571 PMC5465443

[29] Franco-Zorrilla JM, López-Vidriero I, Carrasco JL, Godoy M, Vera P, Solano R. DNA-binding specificities of plant transcription factors and their potential to define target genes. Proceedings of the National Academy of Sciences. 2014;111(6):2367–72. 10.1073/pnas.1316278111 PMC3926073 24477691

[30] Qiu Y-Q. KEGG pathway database. Encyclopedia of Systems Biology. 2013:1068–9.

[31] Miranda RG, McDermott JJ, Barkan A. RNA-binding specificity landscapes of designer pentatricopeptide repeat proteins elucidate principles of PPR–RNA interactions. Nucleic acids research. 2017;46(5):2613–23. 10.1093/nar/gkx1288 PMC5861457 29294070

[32] Jhuang HY, Lee HY, Leu JY. Mitochondrial-nuclear co-evolution leads to hybrid incompatibility through pentatricopeptide repeat proteins. EMBO reports. 2017;18(1):87–101. 10.15252/embr.201643311 27920033 PMC5210125

[33] Datta R, Chamusco KC, Chourey PS. Starch biosynthesis during pollen maturation is associated with altered patterns of gene expression in maize. Plant Physiol. 2002;130(4):1645–56. 10.1104/pp.006908 12481048 PMC166680

[34] Liu Z, Shi X, Li S, Zhang L, Song X. Oxidative stress and aberrant programmed cell death are associated with pollen abortion in isonuclear alloplasmic male-sterile wheat. Frontiers in plant science. 2018;9 10.3389/fpls.2018.00009 29780399 PMC5945952

[35] Koringa P, Jakhesara S, Bhatt V, Meshram C, Patel A, Fefar D, et al Comprehensive transcriptome profiling of squamous cell carcinoma of horn in Bos indicus. Veterinary and comparative oncology. 2016;14(2):122–36. 10.1111/vco.12079 24314272

[36] Wu Y, Min L, Wu Z, Yang L, Zhu L, Yang X, et al Defective pollen wall contributes to male sterility in the male sterile line 1355A of cotton. Scientific reports. 2015;5:9608 10.1038/srep09608 26043720 PMC4456728

[37] Suzuki H, Rodriguez-Uribe L, Xu J, Zhang J. Transcriptome analysis of cytoplasmic male sterility and restoration in CMS-D8 cotton. Plant cell reports. 2013;32(10):1531–42. 10.1007/s00299-013-1465-7 . 23743655

[38] Brown GG, Formanová N, Jin H, Wargachuk R, Dendy C, Patil P, et al The radish Rfo restorer gene of Ogura cytoplasmic male sterility encodes a protein with multiple pentatricopeptide repeats. PlJ. 2003;35(2):262–72. 10.1046/j.1365-313x.2003.01799.x 12848830

[39] Zhao C, Zhao G, Geng Z, Wang Z, Wang K, Liu S, et al Physical mapping and candidate gene prediction of fertility restorer gene of cytoplasmic male sterility in cotton. BMC genomics. 2018;19(1):6 10.1186/s12864-017-4406-y 29295711 PMC5751606

[40] Scott RJ, Spielman M, Dickinson HG. Stamen structure and function. The Plant Cell. 2004;16(suppl 1):S46–S60. 15131249 10.1105/tpc.017012 PMC2643399

[41] Engelke T, Hülsmann S, Tatlioglu T. A comparative study of microsporogenesis and anther wall development in different types of genic and cytoplasmic male sterilities in chives. Plant Breeding. 2002;121(3):254–8.

[42] Piffanelli P, Ross JH, Murphy D. Biogenesis and function of the lipidic structures of pollen grains. Sex Plant Reprod. 1998;11(2):65–80.

[43] Blackmore S, Wortley AH, Skvarla JJ, Rowley JR. Pollen wall development in flowering plants. New Phytol. 2007;174(3):483–98. 10.1111/j.1469-8137.2007.02060.x 17447905

[44] Li D-D, Xue J-S, Zhu J, Yang Z-N. Gene Regulatory Network for Tapetum Development in Arabidopsis thaliana. Frontiers in plant science. 2017;8:1559 10.3389/fpls.2017.01559 28955355 PMC5601042

[45] Higginson T, Li SF, Parish RW. AtMYB103 regulates tapetum and trichome development in Arabidopsis thaliana. PlJ. 2003;35(2):177–92. 10.1046/j.1365-313x.2003.01791.x 12848824

[46] Ma H. Molecular genetic analyses of microsporogenesis and microgametogenesis in flowering plants. Annu Rev Plant Biol. 2005;56:393–434. 10.1146/annurev.arplant.55.031903.141717 15862102

[47] Zhang W, Sun Y, Timofejeva L, Chen C, Grossniklaus U, Ma H. Regulation of Arabidopsis tapetum development and function by DYSFUNCTIONAL TAPETUM1 (DYT1) encoding a putative bHLH transcription factor. Development. 2006;133(16):3085–95. 10.1242/dev.02463 16831835

[48] Feng B, Lu D, Ma X, Peng Y, Sun Y, Ning G, et al Regulation of the Arabidopsis anther transcriptome by DYT1 for pollen development. The Plant Journal. 2012;72(4):612–24. 10.1111/j.1365-313X.2012.05104.x 22775442

[49] Gu JN, Zhu J, Yu Y, Teng XD, Lou Y, Xu XF, et al DYT 1 directly regulates the expression of TDF 1 for tapetum development and pollen wall formation in A rabidopsis. The Plant Journal. 2014;80(6):1005–13. 10.1111/tpj.12694 25284309

[50] Zhu E, You C, Wang S, Cui J, Niu B, Wang Y, et al The DYT 1‐interacting proteins b HLH 010, b HLH 089 and b HLH 091 are redundantly required for A rabidopsis anther development and transcriptome. The Plant Journal. 2015;83(6):976–90. 10.1111/tpj.12942 26216374

[51] Nadeau JA. Stomatal development: new signals and fate determinants. Current opinion in plant biology. 2009;12(1):29–35. 10.1016/j.pbi.2008.10.006 19042149 PMC2645895

[52] Zhang ZB, Zhu J, Gao JF, Wang C, Li H, Li H, et al Transcription factor AtMYB103 is required for anther development by regulating tapetum development, callose dissolution and exine formation in Arabidopsis. PlJ. 2007;52(3):528–38. 10.1111/j.1365-313X.2007.03254.x 17727613

[53] Xu J, Ding Z, Vizcay-Barrena G, Shi J, Liang W, Yuan Z, et al ABORTED MICROSPORES acts as a master regulator of pollen wall formation in Arabidopsis. The Plant Cell. 2014;26(4):1544–56. 10.1105/tpc.114.122986 24781116 PMC4036570

[54] Sella Kapu NU, Cosgrove DJ. Changes in growth and cell wall extensibility of maize silks following pollination. JExB. 2010;61(14):4097–107. 10.1093/jxb/erq225 PMC2935878 20656797

[55] Niu E, Shang X, Cheng C, Bao J, Zeng Y, Cai C, et al Comprehensive analysis of the COBRA-like (COBL) gene family in Gossypium identifies two COBLs potentially associated with fiber quality. PLoS One. 2015;10(12):e0145725 10.1371/journal.pone.0145725 26710066 PMC4692504

[56] Brown DM, Zeef LA, Ellis J, Goodacre R, Turner SR. Identification of novel genes in Arabidopsis involved in secondary cell wall formation using expression profiling and reverse genetics. The Plant Cell. 2005;17(8):2281–95. 10.1105/tpc.105.031542 15980264 PMC1182489

[57] Morant M, Jørgensen K, Schaller H, Pinot F, Møller BL, Werck-Reichhart D, et al CYP703 is an ancient cytochrome P450 in land plants catalyzing in-chain hydroxylation of lauric acid to provide building blocks for sporopollenin synthesis in pollen. The Plant Cell. 2007;19(5):1473–87. 10.1105/tpc.106.045948 17496121 PMC1913723

[58] Li H, Pinot F, Sauveplane V, Werck-Reichhart D, Diehl P, Schreiber L, et al Cytochrome P450 family member CYP704B2 catalyzes the ω-hydroxylation of fatty acids and is required for anther cutin biosynthesis and pollen exine formation in rice. The Plant Cell. 2010;22(1):173–90. 10.1105/tpc.109.070326 20086189 PMC2828706

[59] Han Z, Qin Y, Kong F, Deng Y, Wang Z, Shen G, et al Cloning and expression analysis of eight upland cotton Pentatricopeptide repeat family genes. Appl Biochem Biotechnol. 2016;180(6):1243–55. 10.1007/s12010-016-2164-y 27449222

[60] Kotera E, Tasaka M, Shikanai T. A pentatricopeptide repeat protein is essential for RNA editing in chloroplasts. Nature. 2005;433(7023):326 10.1038/nature03229 15662426

[61] Okuda K, Myouga F, Motohashi R, Shinozaki K, Shikanai T. Conserved domain structure of pentatricopeptide repeat proteins involved in chloroplast RNA editing. Proceedings of the National Academy of Sciences. 2007;104(19):8178–83. 10.1073/pnas.0700865104 PMC1876591 17483454

[62] Chen L, Liu Y-G. Male sterility and fertility restoration in crops. Annual review of plant biology. 2014;65:579–606. 10.1146/annurev-arplant-050213-040119 24313845

[63] Dahan J, Mireau H. The Rf and Rf-like PPR in higher plants, a fast-evolving subclass of PPR genes. RNA biology. 2013;10(9):1469–76. 10.4161/rna.25568 23872480 PMC3858430

[64] Tang H, Luo D, Zhou D, Zhang Q, Tian D, Zheng X, et al The rice restorer Rf4 for wild-abortive cytoplasmic male sterility encodes a mitochondrial-localized PPR protein that functions in reduction of WA352 transcripts. Molecular plant. 2014;7(9):1497–500. 10.1093/mp/ssu047 24728538

[65] Liu Y-J, Xiu Z-H, Meeley R, Tan B-C. Empty pericarp5 encodes a pentatricopeptide repeat protein that is required for mitochondrial RNA editing and seed development in maize. The Plant Cell. 2013;25(3):868–83. 10.1105/tpc.112.106781 23463776 PMC3634694

[66] Li L, Li Y, Song S, Deng H, Li N, Fu X, et al An anther development F-box (ADF) protein regulated by tapetum degeneration retardation (TDR) controls rice anther development. Planta. 2015;241(1):157–66. 10.1007/s00425-014-2160-9 25236969

[67] Zhang Y, Chen J, Liu J, Xia M, Wang W, Shen F. Transcriptome analysis of early anther development of cotton revealed male sterility genes for major metabolic pathways. Journal of Plant Growth Regulation. 2015;34(2):223–32.

[68] Liu C, Ma N, Wang PY, Fu N, Shen HL. Transcriptome sequencing and de novo analysis of a cytoplasmic male sterile line and its near-isogenic restorer line in chili pepper (Capsicum annuum L.). PloS one. 2013;8(6):e65209 10.1371/journal.pone.0065209 23750245 PMC3672106

